# Decreasing serum homocysteine and hypocholesterolemic effects of Bovine lactoferrin in male rat fed with high-cholesterol diet

**DOI:** 10.15171/jcvtr.2018.35

**Published:** 2018-12-11

**Authors:** Samira Nozari, Nazila Fathi Maroufi, Mohammad Nouri, Mirhamid Paytakhti Oskouei, Javad Shiralizade, Farshid Yekani, Mina Mamipour, Yousef Faridvand

**Affiliations:** ^1^Stem Cell Research Center, Tabriz University of Medical Sciences, Tabriz, Iran; ^2^Student Research Committee, Tabriz University of Medical Sciences, Tabriz, Iran; ^3^Stem Cell and Regenerative Medicine Institute (SCARM), Tabriz University of Medical Sciences, Tabriz, Iran; ^4^Department of Biochemistry and Clinical Laboratories, Faculty of Medicine, Tabriz University of Medical Sciences, Tabriz, Iran; ^5^Department of Biochemistry, Faculty of Biology, Payam-e-Noor University of Mashhad, Mashhad, Iran; ^6^Department of Animal Biology, Faculty of Biological Sciences, Kharazmi University, Tehran, Iran; ^7^Department of Biochemistry, Faculty of Medicine, Urmia University of Medical Sciences, Urmia, Iran; ^8^Cardiovascular Research Center, Tabriz University of Medical Sciences, Tabriz, Iran

**Keywords:** Bovine Lactoferrin, High-Cholesterol-Diet, Homocysteinemia, Leptin, Apolipoprotein

## Abstract

***Introduction:*** Lipid metabolism disorder or hyperlipidemia is known as a risk factor for cardiovascular disease, the increase in serum homocysteine and leptin are associated with atherosclerotic disease. The purpose of the present study was to examine the effects of bovine lactoferrin (bLF) on serum homocysteine (Hcy), apolipoproteinA-I (ApoA-I) and B (ApoB), leptin and lipid profile changes in high-cholesterol-diet (HCD) fed rats.

***Methods:*** The Healthy Adult Sprague-Dawley (SD) male rats were randomly assigned into three experimental groups. Each group consisted of eleven male rats including control group, HCD rats and hypercholesterolemic rats, which were treated with bLF (HCD+bLF). bLF was given by gavage (200 mg/kg/d). After 4 weeks of feeding and overnight fasting, total blood samples were collected.

***Results:*** The results showed the elevated level of Hcy, leptin, total cholesterol, low density lipoprotein cholesterol (LDL-C), ApoB and decrease in ApoA-I in non-treated HCD group compared to the control rats. Administration of bLF significantly ameliorated the Hcy and leptin levels with decrease in LDL-C and total cholesterol in rats fed with a high-cholesterol diet. bLF also tended to increase low serum concentration of ApoA-I and high density lipoprotein cholesterol (HDL-C) in HCD rats. Meanwhile, upon bLF-treated rats, there was a significant decrease in ApoB in HCD group.

***Conclusion:*** The findings indicated that bLF can improve the alteration of serum Hcy, leptin, apolipproteins and lipid changes in male rats fed with high-cholesterol diet. So, bLF can counteract with HCD elicited hyper-homocysteinemia and hyper-leptinemia, suggesting it to have the useful therapeutic potential in patients with atherosclerosis and lipid disorder.

## Introduction

Cardiovascular complications resulting from lipid metabolism disorder or hyperlipidemia are known as main cause of death among the patients with coronary heart disease. Hypercholesterolemia is well known as the main risk factor for the development of cardiovascular disease. The accumulation of lipids in the wall of arteries is associated with atherosclerosis occurrence and development. Atherosclerosis is a chronic inflammatory disease associated with lipid metabolism disorder. Excessive production of inflammatory marker and oxidative stress could mediate the chronic disease and are correlated with artery walls damage and the development of atherosclerotic lesions.^1,2^

Homocysteine (Hcy), a thiol-containing amino acid, levels are considered as important indicators of atherosclerosis progression in hypercholesterolemia. Hyperhomocysteinemia is a significant pro-inflammatory factor which has been recognized as a powerful independent risk factor for cardiovascular disease (CVD). The elevated level of Hcy is associated with atherosclerosis lesion and accelerates the plaque formation.^3^ Reduction in serum level of Hcy is warranted because of the direct relationship between plasma Hcy level and the risk of CVDs. Thus, CVD can be prevented if total Hcy levels are reduced^4^ The adipocyte-derived leptin which is primarily involved in the regulation of energy expenditure, exert actions related to cardiovascular homeostasis. Oxidative stress and inflammation are two main causes of CVD .Several studies have demonstrated that leptin contributes to endothelial dysfunction, heart failure and myocardial infarction via the secretion of some pro-inflammatory cytokines and oxidative stress.^5^ The correlation between elevated leptin levels and the formation of reactive oxygen species, fatty acid oxidation and activation of protein kinase A in bovine aortic endothelial cells were indicated by studies.^6^

In recent years, drug treatment or diet therapy has been more focused due to its important role in preventing diseases associated with inflammation, oxidative stress and CVD.^7^ Bovine lactoferrin (bLF), iron-binding glycoprotein is a member of transferrin family with 80 KD weight existing in Whey protein fraction of milk.^8^ bLF receptors on the surface of activated lymphocytes have a mediator role in producing cytokines and reducing the production of nuclear factor-κB (NF-κB), tumor necrosis factor alpha (TNF-α) and increasing interleukin-10. Studies have shown important biological activity of LF including anti-inflammatory, antibacterial, antifungal, anticancer, antioxidant activities and in many biological and chemical environments.^9^ High affinity and reversible binding of bLF to the ferric ion could prevent the production of free radicals such as OH° radicals during the Fenton reaction and thereby prevents the oxidation of lipoproteins.^10^

Currently, we aimed to investigate whether bLF has the protective effect on HCD-induced Hcy and leptin changes that may affect the CVDs and related biomarkers, as well as the apolipoproteinA-I (ApoA-I), apolipoproteinB (ApoB) and lipid profile changes in male rats fed with high-cholesterol diet.

## Materials and Methods

### Animal Treatment and Experimental Design

This study was carried out on 33 healthy male Sprague-Dawley (SD) rats weighting average 180-250 g. Maintenance of rats was carried out under laboratory conditions, 25°C, 48±5% humidity rate and with 12-hour light-dark cycle. Special cages were used to hold the rats with wire tops and plastic bottoms. They were fed with the same type of baits without nutritional limitations. All experimental procedures involving the use of animals approved by the Animal Use and Care Committee of Uremia University of Medical Sciences (Department of Medicine). Rats were randomly divided into three groups (n=11): first group was control group which was fed with normal diet, second group rats were made hypercholesterolemic by feeding with high-cholesterol diet containing 1% cholesterol, 0.05% cholic acid and 5% lard and remaining was normal diet for 4 weeks and finally, last group includes hypercholesterolemia rats treated with bLF (HCD+bLF); (Morinage Milk Industry, Tokyo, Japan) for 4 weeks (200 mg/kg/d dissolved in 0.9% normal saline).^11^ Dose of lactoferrin selected based on published reports of the absence of side effects of lactoferrin values between 0 and 200 mg/kg/d.^12^ bLF was given by gavage (200 mg/kg/d). All rats were sacrificed and their blood was drawn and serums were stored at -70°C for further studies.

### Assessment of serum Hcy and Leptin

The Hcy levels in serum were measured by enzymatic method (Diazyme, USA). First the oxidized Hcy is reduced to free Hcy. In continue reacts with a co-substrate, S-adenosyl methionine (SAM) which is catalyzed by the Hcy S-methyltransferase. This conversion product is amplified by coupled enzymatic cycling reactions. The amount of NADH conversion to NAD^+^ indicated indirectly proportional to the total Hcy level in the sample. A commercial enzyme immunoassay kit (Labor Diagnostika Nord GmbH, Nordhorn, Germany) was used for measuring the amount of leptin.

### Assessment of serum Apo-A-I, ApoB and lipid profile

The analyze of Apolipoprotein A-I (ApoA-I) and apolipoprotein B (ApoB) was done with rat ApoA-I and ApoB ELISA kit (Cusabio Biothech, Wuhan, China) according to the manufacturer’s recommended protocol. The total cholesterol (TC), triglyceride (TG), glucose, high density lipoprotein cholesterol (HDL-C), low density lipoprotein cholesterol (LDL-C) were measured by using the available commercial kit (Pars Azmoun, Tehran, Iran).

### Statistical analysis

SPSS software for Windows (SPSS, Chicago, IL, USA) version 20, was used for statically analyzes. Data was shown as means ± SD. Variance analysis was established by one-way ANOVA. Student’s unpaired *t* test was used to assess the significance of differences between groups (if homogeneity of variables was assumed). A value of *P* < 0.05 was considered as statistically significant.

## Results

Table 1 shows the effect of bLF treatment on serum lipid profile alteration in experimental animals. The cholesterol level of both HCD animals and bLF treated rats was observed to be higher compared to the control rats. This increase in HCD group was significant compared to the control group (*P* < 0.001). Cholesterol levels fed on bLF was observed to be significantly lower (*P* < 0.001) as compared to the HCD rats. HDL-C levels were shown to be significantly increased in bLF group compared to the control group (*P *< 0.001) and HCD group (*P *< 0.05). HCD animals and control group did not show any significant change in the levels of HDL-C. However, bLF group showed higher level of LDL-C compared to control group (*P *< 0.001) but significantly lower level of LDL-C was observed in bLF group as compared with HCD group (*P *< 0.001). The same results were observed for triglycerides and glucose levels. Significantly elevated level in the atherogenic index of HCD group (*P *< 0.001) was observed compared to the bLF treated rats and control group. No significant changes compared to the atherogenic index were observed between control group and bLF rats.

**Table 1:** The Bovine lactoferrin effects on lipid profile in male rats*

Parameter	Control	HCD	HCD+bLF
Final weight (g)	346.36±26.93	547.81±25.19^a^	461.09±25.57^ac^
Glucose (mg/dL)	82.36±11.42	123.45 ± 9.18^a^	105.54 ± 9.71^ac^
Triglyceride (mg/dL)	72.72 ± 8.69	114 ± 9.08^a^	91.90 ± 9.87^ac^
Total-cholesterol (mg/dL)	61.45 ± 8.69	141.36 ± 9.03^a^	103.27 ± 10.29^ac^
LDL-cholesterol (mg/dL)	19.55 ± 2.94	89.73± 10.27^a^	50.09 ± 6.51^ac^
HDL-cholesterol (mg/dL)	28.09 ± 4.57	30.73 ± 6.63	39± 8.03^ad^

^a^ *P* < 0.001; ^b^*P* < 0.05 vs. control rats; ^c^*P* < 0.001; ^d^*P* < 0.05 vs. high-cholesterol-diet (HCD) group. * Experimental groups were fed diets supplemented with 0 mg (control), 1% cholesterol (HCD) and hypercholesterolemia rats treated with 200 mg/kg/d bovine lactoferrin (HCD+bLF). Note: Data are shown as Mean± SD.

**Figure 1:**
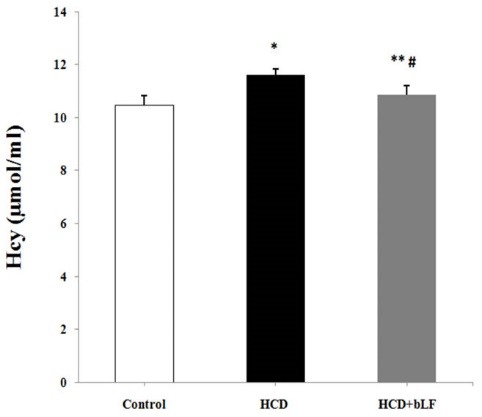
**Levels of homocysteine (Hcy) in bLF group as compared with high-cholesterol-diet (HCD) group and control group;** *Means value was significantly different from control group (11.6±0.24 vs. 10.46±0.36) at *P* < 0.001; **Means value was significantly different from control group (10.87±0.33 vs. 10.46±0.36) at *P* < 0.05; # means value was significantly different from HCD rats (10.87±0.33 vs. 11.6±0.24) at *P* < 0.001; (Mean ± SD).

**Figure 2:**
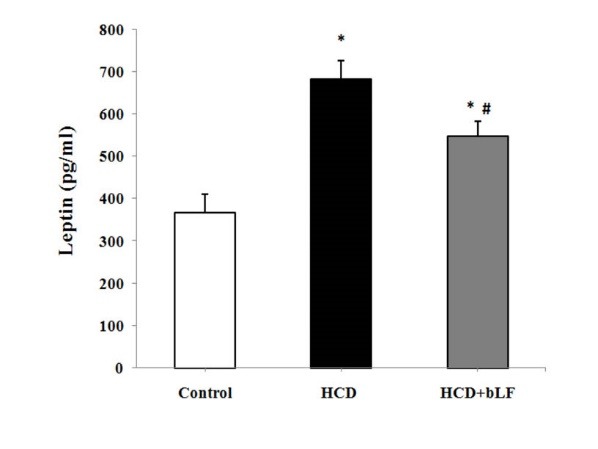
**Levels of Leptin in Bovine lactoferrin (bLF) group as compared with high-cholesterol-diet (HCD) group and control group.** *Means value was significantly different from control group (682.45±43.88 vs. 366.18±43.72; 546.81±35.72 vs. 366.18±43.72) at *P*< 0.001; # means value was significantly different from HCD rats (546.81±35.72 vs. 682.45±43.88) at *P*< 0.001; (Mean ± SD).

Figure 1 shows the decrease in levels of Hcy concentration in experimental groups. Comparing to the control group, the Hcy concentration was significantly increased in the HCD group (*P *< 0.001). Treatment with bLF decreased the Hcy levels in HCD rats compared to the control group (*P *< 0.05). Also, significant decrease in Hcy levels in bLF rats was observed compared to the HCD group (*P *< 0.001). Figure 2 shows the percent changes of leptin concentration in study groups. Feeding with bLF significantly decreased the leptin levels as compared to HCD group (*P *< 0.001).

**Figure 3:**
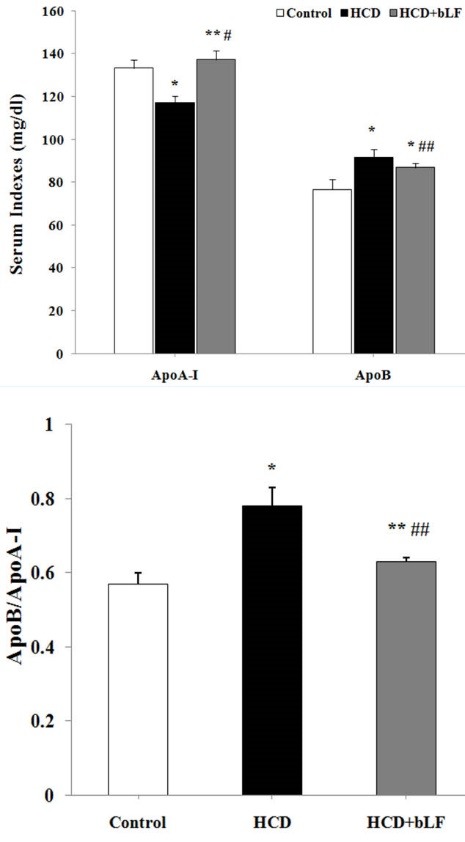
**Effects of the bovine lactoferrin (bLF) on serum apolipoprotein in rats fed with high-cholesterol-diet (HCD).** (**A**) Serum ApoA-I and ApoB levels; (**B**) Serum ApoB/ApoA-I ratio. The data are shown as the mean ± SD. Control: normal diet; HCD: high-cholesterol diet; HCD + bLF: high-cholesterol diet + bovine lactoferrin. ^*^P < 0.001 (vs. control group).^**^P < 0.05(vs. control group). ^#^*P* < 0.001 (vs. high-cholesterol diet rats).^##^*P* < 0.05 (vs. high-cholesterol diet rats); (Mean ± SD).

ApoA-I and ApoB in serum were also measured. Significant reduction of ApoA-I level and increase of serum ApoB level was observed in cholesterol diets (Figure 2). The bLF administration significantly increased the serum ApoA-I level compared with HCD group (*P *< 0.001) and control group (*P *< 0.05). The rise of ApoA-I in HCD+bLF group was statistically significant compared with HCD group (*P *< 0.001). bLF treated group displayed the decreased ApoB levels comparing with HCD group (*P *< 0.05). ApoB/ApoA-I ratio was also calculated in groups and showed that treatment with bLF decreases the ApoB/ApoA-I ratio in HCD+bLF group (Figure 3).

## Discussion

Dyslipidemia is the most important risk factor of atherosclerosis. According to the epidemiological studies, the hyperlipidemia is characterized by hypercholesterolemia and high concentration of LDL-cholesterol (LDL-C) which are important denominators and major factors in atherosclerosis occurrence and development.^13^ In this research, we showed that bLF could decrease serum Hcy and leptin levels which are known as the main risk factors related to atherosclerosis complication. Consistently, bLF could ameliorate serum cholesterol, Triglyceride and LDL-C levels in treatment group. Treatment with bLF associated with increase in serum levels of HDL-C and ApoA-I. Also, ApoB concentration was significantly reduced in male SD rats fed with high-cholesterol diet.

Our data show that bLF administration was effective against hypercholestermia condition. Studies have shown that elevated cholesterol, triglyceride and LDL-C have been associated with CVD.^14^ However, administration of bLF increases the HDL-C and ApoA-I levels rather than reducing the total cholesterol level which indicated the cardio-protective properties of bLF. Researchers have shown that HDL stimulates the reverse cholesterol transport pathway, in which HDL induces efflux of excess accumulated cellular cholesterol and avoids the generation of an oxidative modified LDL.^15^ Badimon et al suggested that HDL administration not only inhibits the progression of atherosclerosis but also reduces atherosclerotic lesions effectively.^16^ Thus, our results suggest that bLF has advantageous effects by promoting efflux of cholesterol accumulated in cells due to increase in the serum concentrations of HDL.

Apolipoproteins, carrier proteins, bind to lipids to form lipoprotein particles and also act as enzyme cofactors and lipid transfer carriers that control and define the metabolic fate of these lipoproteins.^17^ Generally, ApoB transfers lipids from the liver and gut to tissues that use lipids, while ApoA facilitate reverse lipid transport and carries extra lipids from peripheral tissues to the liver.^17^ In the current study, the significant difference was observed between the ApoA and apoB levels in the HCD group with the control group and bLF rats; ApoB levels and the ApoB/ApoA-I ratios were significantly higher and ApoA levels were significantly lower in the HCD group than those in the control group and bLF treated rats. As discussed by Walldius and Jungner, there are benefits in measuring ApoB and ApoA. A high ApoB/ApoA ratio indicated a high number of atherogenic lipoprotein particles, which are placed in the arterial wall. The concentrations of ApoA and Apo-B show the number of their respective lipoprotein particles and the opposite aspects of risks.^18^ Khadem-Ansari et al also described that the serum ApoA-I and ApoB levels are independent risk factors for coronary artery disease (CAD). They recommended the measurement of ApoB and ApoA-I in order to assess the atherogenic potential of lipid disorders.^19^ Here, we observed that bLF can decrease the elevated apoB levels and ApoB/ApoA ratios in HCD rats.

Studies have shown that high Hcy levels or hyperhomocysteinemia (HHcy) increases the risk for the progression of atherosclerosis.^20^ Hyperhomocysteinemia as an effective pro-inflammatory factor is independent of cardiovascular risk factors.^21^ Recent studies connecting Hcy to arteriosclerotic vascular disease showed that Hcy increment causes a 1.6-fold and a 1.8-fold increase in risk for CAD for men and women.^22^ In this study we observed that rats fed with HCD alone (n = 11) showed a significant increase in serum Hcy when compared with the controls rats. Hypercholesterolemia rats treated with bLF showed a significant reduction in serum Hcy levels compared with rats fed with HCD alone. Our result demonstrated that bLF can decrease Hcy levels and have a useful effect in preventing atherosclerosis.

Atherosclerosis is a chronic inflammatory disease. Excessive production of inflammatory marker and oxidative stress could mediate the chronic disease and is correlated with the development of atherosclerotic lesions.^1^ Leptin, the adipocyte-derived molecule, has an important action to regulate energy balance and metabolism.^23^ Importantly, leptin may also exert actions to stimulate vascular inflammation and oxidative stress which may contribute to pathogenesis atherosclerosis and coronary heart disease.^24^ Our findings have indicated that bLF decreases the leptin levels in hypercholesteromic condition. This reduction in leptin levels may display the anti-inflammatory and antioxidant properties of bLF. Studies have documented that leptin induced the secretion of tumor necrosis factor and interleukins-6 and also increases the accumulation of reactive oxygen species.^25^ Hypercholesterolemia also causes endothelial cell injury that is contributed with up-regulation of some inflammatory factors such as NF-κB, interleukin-6 and TNF-α.^26^ The results of this study were in a parallel with Wang et al study who examined the effect of dietary bLF on antioxidant and performance status in piglet. They also found that treatment with bLF has improved activity and mRNA levels of antioxidant. They indicated the exogenous antioxidant activity of bLF.^27^ Mulder et al reported the antioxidant activity and immune modulating properties of oral bLF supplementation in humans.^28^ Similarly, Konishi et al showed that therapy with bLF is a promising therapeutic approach for suppressing oxidative stress in non-responders to antiviral therapy in patients with chronic hepatitis C virus.^29^ Additionally, studies have shown the significant increase in the Hcy and leptin levels in many diseases such as CVDs and inflammation.^30^ The decrease in serum Hcy and leptin may be correlated to the increase of antioxidant capacity or adjunct toward modulation of immune activity in atherosclerosis-related disease.

In summary, the present study has revealed that bLF can decrease serum Hcy, leptin and several traditional risk factors, which are connected with atherosclerosis development such as TC, TG, LDL-C and ApoB in rats fed with high-cholesterol diet. Importantly, our results demonstrated that bLF was effective in an inhibition of atherosclerosis progression and represent the bLF as a promising component in the prevention of atherosclerosis. One of the limitations during this research was nutrition of lab mice every day through gavage. On the other hand, a large number of animals increased the cost and labor load during the study, which caused the study to be prolonged.

## Ethical approval

The ethical approval for this study was obtained from ethics committee of Urmia University of Medical Sciences, Urmia, Iran.

## Competing interests

None.

## Acknowledgments

The authors wish to thank Tabriz University of Medical Sciences and staff of Biochemistry and Clinical Laboratories for kindly providing and supporting this project. We also appreciate the great support provided by Mr. Masoud Isa Khajelou, the English editor of “Depiction of Health” journal.
